# Rapid Review of “No Surprise” Medical Billing in the United States: Stakeholder Perceptions and Challenges

**DOI:** 10.3390/healthcare11050761

**Published:** 2023-03-05

**Authors:** Cristian Lieneck, Mario Gallegos, Madison Ebner, Hannah Drake, Emma Mole, Kaitlin Lucio

**Affiliations:** School of Health Administration, Texas State University, San Marcos, TX 78666, USA

**Keywords:** surprise billing, out-of-network, No Surprises Act, medical dispute resolution, arbitration

## Abstract

Surprise medical bills received after care delivery in both emergency and non-emergency situations for out-of-network (OON) or other contractual health plan regulations adds additional stress upon the care guarantor, most often the patient. The passing and continued implementation of the federal No Surprises Act (NSA) and related state-level legislation continues to influence the processes of care delivery in the United States. This rapid review evaluated the literature specific to surprise medical billing in the United States since the passing of the No Surprise Act, guided by the preferred reporting items for systematic reviews and meta-analyses (PRISMA) protocol. A total of 33 articles were reviewed by the research team and the results demonstrate industry stakeholder perceptions related to two primary industry themes (constructs) surrounding surprise billing: healthcare stakeholder perspectives and medical claim dispute (arbitration) processes. Further investigation identified sub-constructs for each: the practice of balance-billing patients for OON care and healthcare provider, and facility equitable reimbursement challenges (primary theme 1), and arbitration observations and challenges surrounding (a) the NSA medical dispute process, (b) state-level arbitration processes and perceptions, and (c) use of the Medicare fee schedule as a benchmark for arbitration decisions (primary theme 2). The results indicate the need for formative policy improvement initiatives to address the generation of surprise billing.

## 1. Introduction

### 1.1. Introduction to the Problem

The United States (US) healthcare system continues to be challenged by the high costs of care. These costs are experienced by all healthcare stakeholders involved, including the patient, the provider, and the healthcare organization. Often, patients and/or their payment guarantor (third-party insurers/payers) are ‘surprised’ by healthcare facility or provider bills after the delivery of care has ended. Such surprises continue to spur conversation and effort to alleviate financial burdens after the delivery of care by industry leaders, and now federal and state legislators. These surprises are known to occur for both emergency and routine/non-emergency healthcare services and are usually related to care provided behind-the-scenes for the patient, or otherwise indirect care.

### 1.2. Price Transparency Initiatives and Balance-Billing Issues

While price transparency continues to be addressed with ongoing initiatives as related to shoppable services, either online or at the site of care delivery [1], efforts are not directly related to the occurrence of surprise medical bills in our industry. Most often, third-party payer (insurance) organizations credential both medical organizations and providers, listing them as ‘in-network’ with their insurance plan and eligible for allowed (prospective) reimbursement for services provided. However, often, an out-of-network medical provider, usually engaged as an ancillary or behind-the-scenes service, also participates in the patient’s delivery of care and, as a result, an out-of-network (OON) medical claim is generated [1,2]. These OON claims are not based upon a third-party payer’s allowed amount set forth by prospective fee schedules and, therefore, do not include a contractual adjustment. As a result, the OON claim is due by the patient receiving care [2].

Additionally, if a third-party payer processes an OON medical claim and permits some reimbursement to the medical provider and/or healthcare organization, any difference between such reimbursement and the billed charge is often balance-billed to the patient, due upon receipt. Again, processed as OON care, no contractual agreement or allowed amount fee schedule exists and the provider’s full billed charge for care will need to be paid eventually.

### 1.3. No Surprises Act

Effective from 1 January 2022, the federal No Surprises Act (NSA) disallows any surprise OON medical bill (at the full charge, OON rate) for patients receiving care without prior authorization from the OON provider, any OON cost sharing (to include coinsurance and/or copayment amounts for emergency and some non-emergency services), and behind-the-scenes care such as radiology, laboratory, or anesthesiology (for example) [3]. While most surprise billing occurrences are experienced by patients with third-party health insurance, uninsured patients may also experience similar surprise medical bills [4].

### 1.4. NSA and State-Level Dispute Resolution Procedures

Many states in the US previously adopted no surprise legislation at the state level. Both state legislation and now the federal NSA address the inability of a medical provider to balance-bill an insured patient for OON care provided without prior notice [5]. However, exceptions and other situations do still occur, and disagreements on the allowed amount and/or expected reimbursement rate to be paid to the hospital and/or medical provider continue to exist. As a result, dispute resolution processes have been established to help resolve disagreements in the reimbursement of care due to providers and facilities. [5]

### 1.5. Arbitration and Related Medical Dispute Resolution Processes

Independent Dispute Resolution (IDR) is the process used per the federal NSA to address any disagreement in amount due for care as a result of a surprise medical bill. This is an arbitration approach where a dispute is submitted, and an arbitrator makes a binding decision on the matter in the end. Here, both parties (often the insurer and the provider/organization) offer an amount to be paid and one side is selected by the arbiter [5,6]. There is no other method to establish the amount to be paid; therefore, either side will result in a financial win and/or loss per the new policy [6]. Industry concerns of potential price inflationary actions, an inability to cover cost of care provided, and a potential motivation for providers to remain OON to retain the ability to charge higher rates continue to be of concern.

To date, limited research has been conducted to investigate the industry perceptions and challenges related to surprise billing and the federal No Surprises Act (NSA) and ongoing state-level legislation. While financial studies imply the potential for industry challenges and benefits from the legislative efforts, further understanding of the application of policy upon the industry and those receiving care is needed to address potential policy implications and contribute to the policy revision process. To our knowledge, no rapid systematic literature review surrounding perceptions and observations of surprise billing associated with the NSA has been conducted to date. This review initiative attempts to provide additional insight into this gap in the literature as policy implementation continues and healthcare stakeholders are affected.

## 2. Materials and Methods

### 2.1. Overview

The research team’s intent was to specifically investigate surprise billing concepts and related perceptions by industry stakeholders in the United States present in the literature guided by the PRISMA review standard. Four main research databases were queried using the EBSCOhost platform via the Texas State University’s library website: the Cumulative Index to Nursing and Allied Health Literature, PubMed/MEDLINE, Complementary Index, and Academic Search Complete.

### 2.2. Inclusion Criteria

Study search terms were carefully chosen for this study based upon the proper identification of current publications/research surrounding the surprise billing policy, stakeholder perceptions, and/or other related topics. The research team initially reviewed the listed MeSH (Medial Subject Headings) controlled vocabulary thesaurus, utilized for indexing articles for PubMed. However, no specific ‘surprise billing’ and/or related terminology was identified in this resource. As a result, typical healthcare industry billing and reimbursement terminology provided too broad a database query, not specific to the team’s “surprise” billing topic.

As a result, the research team utilized Google search queries to establish a search staring terminology and Boolean operators that identified applicable publications surrounding the research topic. The final search string utilized was:

[(“surprise*”)] AND [(“bill*”) OR (“No Surprises Act”) OR (“Consolidated Appropriations Act”)]

Initial search results were 132,269 articles and, after filtering for publication dates between 1 January 2021 and 5 July 2022, the research team identified 7709 articles. The 1 January 2021 date was utilized in the search criteria based upon the US Congress passing of the CAA of 2021 (which included the No Surprises Act) on 27 December 2020, with an implementation date of 1 January 2021 to the current date of the research team’s investigation (5 July 2022).

### 2.3. Exclusion Criteria

Published articles were included in this review if eligible surprise billing was specifically addressed as either the publication’s main topic, or potentially as an underlying theme within any identified article. Publications included in the review analysis had to be reported in quality journals (peer-reviewed) and also meet the January–July publication date range. The review team immediately recognized that publications identified in the initial database queries did not address healthcare industry outcomes as related to surprise billing identified instances. Therefore, the team’s research objective was to focus primarily on surprise billing perceptions by industry stakeholders and related sub-topics later identified during the review/analysis process.

**Figure 1:**
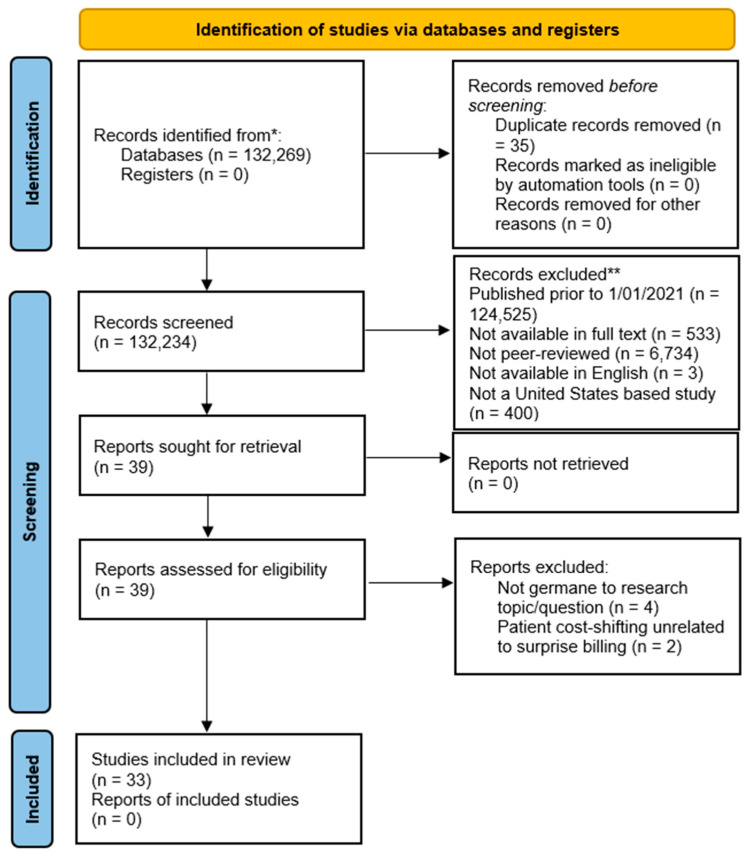
Preferred reporting items for systematic reviews and meta-analysis (PRISMA) figure that demonstrates the study selection process.

Additional database search engine parameters were applied to produce focused, applicable results to meet the research objective. The EBSCOhost database platform automatically removed 35 duplicate publications from the initial search query. In additional to filtering for publication date, the research team further excluded any publications that were not available in full-text format (−533 articles); were not published in peer-reviewed publication outlets (−6734 articles); were not available in the English language (−3 articles); and not a United States-based study (−400 articles). These exclusion steps were conducted using the EBSCOhost platform’s available menu/check-box filter options before any abstract and/or full text investigation initiated and yielded 39 remaining articles. Figure 1 illustrates the research team’s rapid review process and the applied search exclusion criteria.

A review of the studies included in this review was conducted by the authors, to include full manuscript review with each identified publication being reviewed by at least two members of the research team. Table 1 shows the delineation of three sets of 11 review articles assigned to the research team members.

The research team’s full text review for eligibility resulted in 33 remaining publications remaining in the review. Six of the 39 articles were removed from the review by the research team as follows:

- Four articles were deemed not germane to the research topic. Either erroneously added by the initial database search, or somehow mentioning “surprises” in healthcare delivery, yet not related to “surprise billing” practices and/or perceptions.
- Two articles were removed for focusing on themes unrelated to this study, yet still mentioning “surprise billing.” While briefly mentioned, the articles were not addressing surprise billing specifically but rather just listing it as a potential reason for a variety of patient cost-shifting methods occurring in the industry.

The research team collaborated both in-person and online via webinars to address any potential article bias or conflict with the application of the selection criteria for the review. Several consensus meetings resulted in no disagreement among the research team members in this regard.

## 3. Results

### 3.1. Study Characteristics

The team’s full-text review of the 33 articles identified underlying constructs (characteristics) associated with surprise billing (perceptions and/or challenges) within the US healthcare system. A summary of review findings for each article is provided in Table 2.

### 3.2. Identification of Underlying Constructs

**Figure 2:**
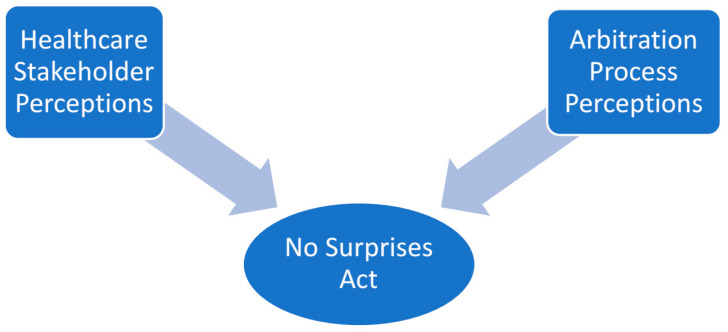
Primary occurrences of surprise billing underlying themes (constructs) identified in the literature.

Early in the review team’s consensus meetings, two primary themes were identified in the literature, supporting the research topic of perceptions and challenges related to the occurrence of surprise billing in the United States (Figure 2).

**Figure 3:**
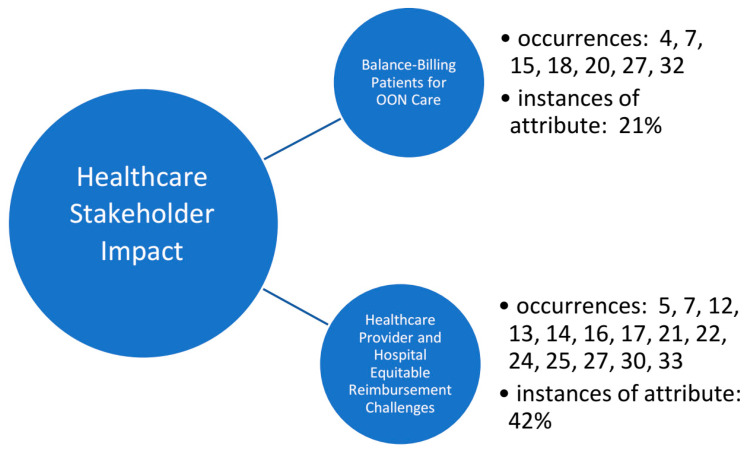
Occurrences of surprise billing underlying stakeholder themes (constructs) identified in the literature and supporting metadata.

Further review and analysis supported the investigation into the impact and related perceptions of surprise billing upon various healthcare industry stakeholders. Initially the research team proposed delineating the impact of surprise billing by stakeholder type (patient, provider, and healthcare insurance company/third-party payer). However, ongoing analysis and discussion by the review team resulted in dichotomous, financially related sub-topics, centered around the healthcare organization’s balance-billing of patients for out-of-network (OON) care and reimbursement challenges for medical providers and hospital organizations (Figure 3). Findings are not mutually exclusive to either sub-construct identified below, as several articles supported each theme.

**Figure 4:**
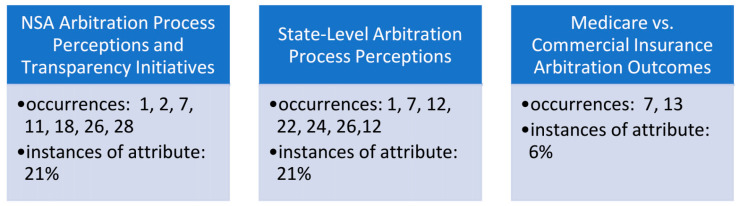
Occurrences of surprise billing underlying arbitration themes (constructs) identified in the literature and supporting metadata.

Healthcare provider and hospital reimbursement challenges were identified to focus specifically on the topic of medical claims arbitration and related observations of various independent dispute resolution (IDR) perceptions within the industry. This construct was deconstructed into three sub-constructs: the No Surprises Act processes and transparency initiatives; state-level arbitration process perceptions; and observations related to the comparison of the Medicare programs’ arbitration outcomes, as compared to commercial insurance arbitration practices and outcomes (Figure 4). Findings are not mutually exclusive to either sub-construct identified below, as several articles supported each theme.

## 4. Discussion

### 4.1. Summary of Evidence: Healthcare Industry Stakeholder Perceptions of the No Surprises Act

The US government’s initiative to monitor and support transparency and price control within the healthcare industry continues to challenge all stakeholders, including patients, providers, and organizations. As with any policy implementation process, unintended consequences during implementation phases will often occur and these experiences and related information are vital to ensure quality improvement goals. Evidence from this review suggests that US healthcare system stakeholders are all affected by the occurrence of surprise billing and the No Surprises Act in one primary way—financially. Individuals seeking medical care may experience medical bills after seeking care, associated with balance-billing for OON services rendered—often without their knowledge of the OON medical provider participating in their care in the first place [10,13,21,38].

Additionally, healthcare provider and organization reimbursement challenges will continue to relate to and rely upon an arbitration process (or processes), also often occurring long after the provision of medical care has ended [18,27,33]. A majority of the articles discussed the No Surprises Act addressing the healthcare market failure of billing patients for out-of-network charges or services, even in situations of receiving care at an in-network facility in which employers and insurers often absorb the cost if an OON provider is involved in the treatment of the patient.

#### 4.1.1. Stakeholder Impact: Balance-Billing Patients for OON Care

The research team identified surprise billing to be experienced by the patient primarily as a result of care received from OON medical providers (and/or medical facilities) [10,21,38]. Such care was most often related to patients requiring emergency care, often where consent for care was implied [10,24,34]. These situations could involve the patient having an altered or unconscious mental status, respiratory distress, or even trauma. As a result, the recent need for federal healthcare regulation was identified and implemented via the No Surprises Act when it comes to billing additional amounts to healthcare consumers for OON care, especially for emergency situations [13,38]. Even if conscious and alert, patients in similar situations have been cited as experiencing this problem of OON care and related unaffordability/financial concerns [26].

The research team also identified several articles explaining the patient experiences of OON care and surprise billing as related to mental health concerns after initial care has been completed. Unexpected medical bills has been cited as a leading cause for stress among insured Americans, compromising up to two-thirds of adults [10,26]. On average, almost 20% of emergency department visits result in at least one surprise bill to the patient afterward, and this rate varies by state [10,13,26]. In Texas, California, Florida, Kansas, New York, and Washington, the probability of receiving a surprise medical bill is closer to 30% [13,38]. Even OON care that is delivered in a non-emergent, or on a routine basis occurs. An example includes specialty medical provider services that tend to surprise and balance-bill more often compared to other medical services, such as out-of-network claims for insured patients having a colonoscopy [38].

#### 4.1.2. Stakeholder Impact: Healthcare Provider and Hospital Equitable Reimbursement Challenges

A hospital’s ethics committee may decide whether a physician can set and charge a fee as an OON provider involved in the process of care. For example, hospital ethics committees are often asked to consider if unanticipated billing of a patient for the cost of healthcare services that are not covered by the patient’s health insurance by physicians in the hospital emergency department (ED) is unethical when the patient has contractual in- network coverage for the hospital itself [39]. Here, the American Medical Association (AMA) Code of Ethics provides some advice, stating that while patients must pay their financial responsibilities regardless of hardships, the provider’s fee must be reasonable and not excessive [39]. This becomes a complicated situation, where often the medical provider is not employed by the hospital and is therefore billing the professional component of the service separately from the hospital’s usual and customary charges (technical component).

Alternatively, while medical providers and organizations are to now disclose any financial and other factors that could affect the patient’s care, this price transparency initiative has been observed as both complicated and flawed in the US. [1]. Some emergency department providers are independently contracted, also common with anesthesiology and radiology services. Although the hospital may disclose to the patient that the emergency room physician services are independently contracted, under the Federal Emergency Medical Treatment and Labor Act (EMTALA), such financial discussions do not occur before a patient is screened [38]. To legislatively regulate balance-billing, the AMA and AHA (American Hospital Association) hold that patients should not be balance-billed for emergency services or for out-of-network services obtained in any in-network facility when they could have assumed the providers were in-network with their health plan [38]. These situations and resulting policies make medical provider and hospital reimbursement challenges even greater and less equitable as compared to those patients receiving 100% in-network care.

Effective this past January 2022, the No Surprises Act affects who and how out-of-network rates will be set under the new legislation [13,18]. Further, in 2018 the state of New Jersey implemented an arbitration system to create a solution for surprise medical bills and disputes between patient and out-of-network providers. Another study demonstrated that the billed and paid prices paid to providers dropped significantly after the implementation of the federal No Surprises Act [13,18,27]. To compromise between fairness to the patient and insurers, as well as the providers, and arbitration “final offer” process has been set in place to avoid pricing extremes. The implications of this law have continued to be cited as negatively affecting physician revenues, while increasing pricing transparency, and dropping commercial insurance premiums [18,20,27,34].

### 4.2. Summary of Evidence: Surprise Billing Arbitration Themes

In further review of the literature, the research team identified several arbitration-related topics and sub-themes. These observations, often presented from the viewpoint(s) of various stakeholder perceptions in the articles, focused on the financial outcomes and experiences as related to the arbitration processes and transparency of such outcomes, various state-level arbitration regulations and related financial outcomes and perspectives, and also articles addressing a comparison of financial equity by comparison of arbitration outcomes for Medicare beneficiary claims versus that of commercial insurance claims.

#### 4.2.1. No Surprise Act Arbitration Process Perceptions and Transparency Initiatives

The research team was overwhelming convinced that the dispute resolution process with healthcare financial reimbursement challenges for patients, providers, and healthcare organization existed as a primary theme in the literature surrounding surprise billing. No Surprise Act literature is particularly useful in understanding some of the aspects of the new legislation, including who it affects, what political ramifications it has, and how out-of-network prices will be determined under the new legislation [14,24]. While most of the articles in the review cited the term(s) dispute resolution (or independent dispute resolution) and/or arbitration, the team chose to identify and categorize those specifically focusing on arbitration processes, procedures, perspectives—and especially financial outcomes as experienced by the healthcare stakeholder.

Hall [24] specifically completes this task, therefore addressing perspectives on the claim arbitration process when surprise billing occurs after care delivery. While most perspectives follow in line with the arbitration outcome and financial benefit outcome (or loss) by stakeholder, the equity of reimbursement to the medical provider and/or healthcare organization remains of concern [8,17,24]. Additionally, the literature suggests that after the No Surprises Act was enacted, new price sharing rules, arbitration agreements, and policy implementation will continue to be required to properly close the payment gap produced by billed services and transparency concerns [17]. The state of Texas preempted the No Surprises Act, addressing the Texas Medical Association’s (TMA) measures taken before and in response to the federal government’s decisions [7,8].

The arbitration processes and procedures between insurers and providers and/or organizations are thoroughly described in the literature [7,32], while areas for improvement and suggested change continue to exist. Cited as the “gold standard,” the state of Texas Senate Bill 1264 focused on surprise billing and is often compared to the US federal policy and related implementation efforts [13]. As a result, this state’s process is often duplicated and/or used as a template for other states to follow in this regard. Further, it was identified in the literature that all surprise billing legislation will only continue to reduce “competitiveness… reduce consumer cost… [and] add regulatory complexity for some physician practices; for patients” [34]. Additionally, the articles do continue to address how this legislation continues to create a market of fairness and transparency in the end [6,13,17,34].

#### 4.2.2. State-Level Arbitration Process Perceptions

Many states such as Florida, California, Texas, and New York had prior legislation (statewide) in place addressing surprise billing before the No Surprises Act was passed at the federal level [13,18,30]. Prior to the No Surprises Act passing at the federal level, a total of 29 states already had established legislation to address surprise billing regulations, varying in scope [18,34]. Inherent to the federalism process in the US, therefore granting states the power to pass their own policies surrounding local challenges and needs, many states’ healthcare facilities preferred their particular state arbitration method compared to the recent federal legislation [18,28]. This was most often cited due to state law being more inclusive to state needs, not broad, national policy and related federal needs [7,13,18,34].

For instance, New York’s solution to surprise billing discrepancy is arbitration between provider and insurers [32]. Resolution systems that rely on arbitration raise healthcare costs and are favored by physicians and hospitals [32]. Another common state approach is benchmarking; benchmarking limits out-of-network charges to a percentage of the in-network discounted price of the service provided. Both insurers and employers favor this approach and have been proven to push prices down and slow inflation by imposing clear price limits [7,13,32].

Overall, the federal legislation was well intended, addressing the need to end patient burden through surprise billing. The legislation, highlighted through state programs, shows the lack of federal initiative to make payment fair to both the provider and the patient at times. Reimbursement is a critical talking point at both the state and federal levels surrounding arbitration processes and outcomes, and the nation will likely see future amendments to address them.

#### 4.2.3. Commercial Insurance Arbitration Final Offers versus Medicare Allowable

The research team identified that arbitrators adjudicating out-of-network payment disputes under the No Surprises Act often award (on average) about 314% higher allowed payment for commercial insurance dispute resolution compared to traditional Medicare allowed reimbursement [19]. This observation (arbitration results from out-of-network claim disputes) is not uncommon and seen in multiple studies to date [13,19]. Although specific to anesthesiology services for hospitals, this finding demonstrates a disparity between arbitration outcomes for similar services taken into dispute resolution processions by the payer. However, between both payer-types (Medicare versus commercial insurance), the literature cited an inherent motivation for medical providers to remain out-of-network in an attempt to secure higher third-party reimbursement [19]. Such observation was provided and applied to regularly processed claims, as well as those coming out of arbitration processes [13,19].

While an option to balance-bill the Medicare beneficiary does not exist, the use of allowed amounts—as utilized appropriately with applicable conversion factors—did seem appropriate and feasible [13]. Providers and organizations may utilize Medicare’s published reimbursement fee schedule to benchmark arbitration outcome successes and/or failures from a reimbursement standpoint. While unique variables do exist, it does allow for external benchmarking analyses.

The state of New Jersey’s arbitration system was assessed with Medicare claims’ adjudication processes and outcomes, yielding multiple recommendations for guiding future legislation around surprise billing initiatives and the known (or unknown) provision of out-of-network care [19]. Specifically, Chartock et al. [19] provide further details, demonstrating a 5.7 prevailing median in-network rate of the 18th percentile of provider charges for similar services that arbitrators are awarding for the state. Additionally, as follow-on repercussions of no surprise legislation at both federal and even state (New Jersey) levels, it is suggested that bargaining levels with commercial insurers decrease, as well as a potential increase to healthcare costs as a result of arbitration results compared to Medicare allowable rates and even dispute administrative costs [19]. Inherent in all these processes is the initiative for medical providers and organizations to increase their prices for healthcare services over time.

## 5. Conclusions

The United States healthcare industry continues to struggle with third-party payer contractual network agreements and related reimbursement challenges. As long as medical providers and their organizations are categorized into in- and out-of-network contractual arrangements, surprise medical bills will continue to be generated based upon the fragmented structure of care delivery in the United States.

This systematic review identified perceptions and challenges experienced by industry stakeholders (patients, providers, insurers, and care organizations) since the recent passing of the NSA, implemented in January 2022. Such stakeholder observations were able to be sub-categorized into the balance-billing of OON for patients, as well as hospital and provider reimbursement equity challenges as a result of the adapted arbitration processes. Additionally, the arbitration process (dispute resolution) was able to be assessed into the process itself and related industry stakeholder perceptions regarding the process, state-level arbitration practices, and use of the Medicare fee schedule in arbitration proceedings and arbiter decisions.

Several limitations were identified in this review and, therefore, offer additional areas for future research and investigation. A majority of the articles identified by the PRISMA review process (method) are qualitative in nature and provide descriptive experiences of patients and other third-party guarantors receiving surprise bills for medical services provided. The review process involved a research database search that included articles published within a specific time period, up to the date of the researcher team’s effort to conclude the search for potential articles on surprise billing and begin the manuscript review and analysis steps. However, the surprise billing topic is quite dynamic, and ongoing publications and potentially new constructs (themes) may be identified with updated systematic reviews and related research efforts. Limitations of the systematic literature review methodology include the date range of manuscripts included in the review process, time between the database search process, and the review team’s analysis/write-up of findings and publication. The review’s methodology was only able to identify 33 articles on the topic to date at the time of the database search. Finally, ongoing changes with such a dynamic industry subject may be addressed within updated and/or completely new systematic literature reviews.

Future research surrounding the NSA and related industry perceptions and challenges may involve assessment of ongoing NSA updates and policy amendments (which continue to occur), and additional/other attempts to map arbitration decision outcomes to practical and usual/customary fee schedules without influencing price inflationary practices by the provider and/or healthcare organization.

## Figures and Tables

**Table 1:** Reviewer assignment of the initial database search findings (full article review).

Article Assignment	Reviewer 1	Reviewer 2	Reviewer 3	Reviewer 4	Reviewer 5	Reviewer 6
Articles 1–11	X	X	X	X		X
Articles 12–22			X	X		X
Articles 23–33					X	X
Articles 41–50				X	X	X

**Table 2:** Summary of findings (*n* = 33).

Author(s)/Article Number	Title	Publication Name	Participant(s)	Surprise Billing Perceptions/Observations
Berlin, J. [7]	Federal Fairness? Congressional Measure Addresses Out-of-Network Payments	Texas Medicine	State of Texas patients and medical providers	The State of Texas passed its own state-level Senate Bill (SB) 1264 in 2019 to address surprise billing for out-of-network care provided to patients. The main purpose of this article was to highlight the “gold standard” of the bill SB 1264 that was implemented in Texas before federal legislation in the No Surprises Act (2022) was implemented. The author concludes that SB 1264 in Texas is fair to both the patient and the physician; the author also suggests that the federal law, while off to the right start, should mirror the Texas law when it comes to the arbitration process, payment selection, and payment of the disputed bill.
Berlin, J. [8]	Making Billing More Balanced: Congress Considers Surprise Billing Legislation	Texas Medicine	State of Texas, New York, and California medical providers and professional stakeholder associations and related stakeholders	Out-of-network care payment disputes should not include the patient. Reimbursement to the medical provider should be fair. IDR should be a fair process that is not biased to either the medical provider or the insurance company, while records of the medical provider threatening future IDR has resulted in increased reimbursement beyond in-network rates. New York State’s IDR process is cited by insurance companies as threatening to their solvency, while California’s process is threatening to physicians because it utilizes a median in-network rate for IDR resolution. The article also addresses the Texas Medical Association’s (TMA) measures taken in response to the federal government’s decisions.
Bernstein, J. [9]. Clinical, 478(10), 2213-2217–2217.	Not the Last Word: Surprise Medical Bills are Hardly Charitable	Orthopaedics and Related Research	Assessment of potential surprise billing stakeholders, including patients, providers, and healthcare organizations (hospitals)	Cites three reasons for surprise billing—emergency care, elective non-emergency care, and medical providers—in which the patient did not hire for care directly. The author cites emergency care as the easiest to fix with no surprise billing legislation, and recommends that any not-for-profit hospital absorb the costs of out-of-network ‘behind the scenes’ providers that provided care without prior patient authorization. The author even cites a potential ethical concern where medical providers could intentionally stay out-of-network on purpose and provide emergency care to “captive” patients in need, later balance-billing patients for denied medical claims.
Biener et al. [10]	Emergency physicians recover a higher share of charges from out-of-network care than from in-network care	Health Affairs	Medical providers and patients	Medical Expenditure Panel Survey (MEPS) data were used to assess an estimated amount of out-of-pocket expenditures that patients are burdened with annually. The study found physicians growing revenues by remaining out-of-network at in-network facilities and billing their patients the difference in the covered amount by private insurers; estimations for surprise bills occurring (before the No Surprises Act was at about 5–15% likelihood and overall prevalence at about 4–22%. The high probability of balanced or surprised bills occurring, especially among the emergency department patients, highlights a need for federal balance-billing regulation.
Brown et al. [11]	Out-of-Network Air Ambulance Bills: Prevalence, Magnitude, and Policy Solutions	The Milbank Quarterly	Air Ambulance provider organizations and emergency patients	The issue of surprise billing is cited as an issue with air ambulance emergency response and related care. An inability for the patient to select in- or out-of-network providers exists. Based on the nature of the service provided by air ambulances, 75% of these responses for emergency care are out-of-network and involve balance-billing the patient afterward.
Busch S. & Kyanko, K. [12]	Incorrect provider directories associated with out-of-network mental healthcare and outpatient surprise bills	Health Affairs	Mental health patients receiving care from out-of-network providers	Privately insured patients received outpatient surprise bills as a result of poor experiences with medical provider directories. Many patients became aware that a provider was out-of-network at the first appointment or when they received the bill after the service was provided. Patients with fair or poor self-reported health were significantly more likely to experience surprise bills, and 12% noted that a provider listed in their insurance directory had either incorrect contact information or did not take their insurance. Of visits to out-of-network providers (N = 654), 39% were associated with surprise bills. Most out-of-network visits (70%) were not reimbursed by the insurer, with no out-of-network coverage and an unmet deductible being the most cited reasons. Surprise bills were no more likely to come from specialists compared with primary care providers.
Chartock, B. et al. [13]	Arbitration over out-of-network medical bills: Evidence from New Jersey payment disputes	Health Affairs	State of New Jersey patients and providers involved in the claim arbitration process with commercial insurance companies	The arbitration process in New Jersey is reviewed as a method to resolve the payment disagreements between the out-of-network facility and insurance companies. An analysis compares Medicare versus commercial data claims settled through arbitration by linking administrative data from New Jersey arbitration case outcomes. The study concluded that, if arbitration rates are set off previous provider-billed rates, healthcare costs will continue to increase in the future.
Chhabra, K. et al. [14]	Most patients undergoing ground and air ambulance transportation receive sizable out-of-network bills	Health Affairs		The prevalence and financial impact of out-of-network billing in both ground and air ambulance services exists. Both ground and air ambulance balance-billing for out-of-network care was analyzed by dividing the number of encounters with out-of-network charges. Results demonstrate that the prevalence of surprise bills in ground transportation decreased from 70% in 2013 to 69% in 2017. The frequency of surprise bills in air transportation increased from 60% in 2013 to 71% in 2017. According to this article, this is the first study to document the liability that patients have when insurance plans do not pay the full charge from out-of-network ambulance providers. Air ambulance transportation is cited as declining, while air ambulances charges are rising.
Chhabra, K. et al. [15]	No More Surprises—New Legislation on Out-of-Network Billing	The New England Journal of Medicine	Medical providers, patients, and healthcare organizations in New York, California, and the United States	Evaluation of both New York and California’s state-level balance-billing legislation is conducted with pros/cons highlighted for both. Comparison to federal legislation is also conducted and reimbursement rates are compared for various IDR venues. The author claims that the federal legislation will potentially reduce reimbursements for providers who use surprise billing as a business tactic, such as large physician staffing firms in emergency medicine and anesthesia. Additional work on policy to specifically address ground ambulance surprise billing is also mentioned.
Chhabra, K. et al. [16]	Out-of-Network Bills for Privately Insured Patients Undergoing Elective Surgery with In-Network Primary Surgeons and Facilities	JAMA, The Journal of the American Medical Association	Patients receiving elective surgery and their providers	Surprise billing for elective services is assessed within in-network facilities to insured patients. Both the total out-of-network charges that are less than the typical in-network price and the frequency of out-of-network bills were analyzed in this study. Findings demonstrate that 347,356 patients (20.5%) had received an out-of-network bill. Among these out-of-network bills, 37% that were associated with surgical services listed an average cost of care at $3633, and the calculated potential bill ranged from $1866–$2157. Compared to surgical operation with no complications, those with complication had a significantly higher risk of out-of-network bills.
Colla, C. [17]	Surprise Billing—A Flashpoint for Major Policy Issues in Health Care	JAMA, The Journal of the American Medical Association	Healthcare stakeholder professions associations, medical providers	Transportation is a major issue in surprise billing with 69% of air ambulance involving out-of-network billing. Efforts to stall surprise billing legislation are prevalent today and cited as a result of private equity firm investment in the healthcare industry. In 2018, across hospital inpatient and outpatient services, private insurers paid approximately 2.5 times more than Medicare would have paid for the same services at the same facilities, varying significantly across all states. Policy options have included benchmarking (setting a price cap based on prices in the area) and arbitration. The author cites no surprise billing legislation as a step in the right direction to protect consumers of health services.
Cooper, Z. et al. [18]	Surprise! Out-of-Network Billing for Emergency Care in the United States	Journal of Political Economy	New York state’s no surprises balance-billing initiatives and related stakeholders	By examining New York’s efforts to resolve out-of-network billing through binding arbitration between physicians and insurers over out-of-network payments, this paper demonstrates that having a strong outside choice strengthens physicians’ bargaining position with insurers. Out-of-network billing was lowered by 12.8 percentage points as a result of this intervention. Unfair surprise billing practice in emergency care situations is addressed and certain practices and physician groups deliberately bill out-of-network with the intent to raise their profits are discussed.
Duffy, E. et al. [19]	Commercial and Medicare advantage payment for anesthesiology services	American Journal of Managed Care	Medical provider reimbursement for services provided	A variance in Medicare Advantage payment set rates compared to privately insured (or other patient payment plan) set charges for provider services. A disproportionate rate-setting method is cited to persuade services like orthopedics or anesthetics to remain out-of-network to set their own charges or settle charge disputes. Allowed amount and charges for commercial claims, as compared to Medicare Advantage claims, were used to compare network status and provider reimbursement for services. A variance amongst these pay groups is identified, therefore disincentivizing the nature of in-network status. Surface-level arbitration processes are cited as not being inclusive towards specialists when setting charge rates and billing the customer.
Duffy, E. et al. [20]	Policies to address surprise billing can affect health insurance premiums	American Journal of Managed Care	Commercial health insurance companies and their members/beneficiaries	Increased out-of-network spending is noted as an influence of rising insurance premiums for taxpayers and healthcare consumers. Ancillary spending and its impact on the charges that the services have on health insurance premiums is analyzed using three of the largest healthcare providers—UnitedHealthcare, Aetna, and Humana. Findings suggest that although surprise billings carry a financial burden on the individual, insurance premiums are at least 5% higher than they would be given this market failure. The impact of surprise billing on commercial insurance and, consequently, the premiums associated with the provided health insurance demonstrates that reducing payment for typically balanced-billed services will corelate with lower premiums commercially.
Duffy, E. et al. [21]	Prevalence and characteristics of surprise out-of-network bills from professionals in ambulatory surgery centers	Health Affairs	Ambulatory surgery center provider organizations and patients	4.2 million ambulatory surgery centers’ episodes of care involving patients enrolled in UnitedHealth Group, Humana, and Aetna were analyzed to assess the scale and prevalence of surprise billing. Surprise balance-billing occurrences were identified for ancillary services such as anesthesiology (47%) and nursing care (17%). Policymakers are called on to create legislation to protect commercial consumers from surprise bills; analysis of insurance claims from three of the big private insurers represented a large percentage and prevalence of surprise bills occurring in the study.
Fuse Brown, E. et al. [22]	What States Can Do to Address Out-of-Network Air Ambulance Bills	Journal of Law, Medicine and Ethics	Air ambulance patients and federal/state policymakers	The authors claim that air ambulance surprise bills are particularly unjust due to their ad hoc character, infrequent occurrence, and exorbitant charges. Little legal protection is mentioned for consumers facing out-of-network air ambulance fees. A federal solution is cited as the best option to controlling surprise billing related to air ambulance care. Further, state-level channels of jurisdiction and tools are recommended to protect consumers against out-of-network air ambulance expenses.
Fuse Brown, E. et al. [23]	Stalled Federal Efforts to End Surprise Billing—The Role of Private Equity	The New England Journal of Medicine	US Congress policymakers	Factors associated with surprise billing and the HELP bill failing are discussed. The HELP bill included consumer protections to ensure that consumers would only pay the amount of their in-network bill. Prior efforts by the US Congress to address surprise billing are summarized; yet ultimately shut down due to disputes between other entities prior to successful legislation eventually being passed.
Hall, M. [24]	A “Surprise” Health Policy Legislative Victory	The Hastings Center Report	Healthcare policymakers, medical providers, and insurers	Political clout surrounding the recently passed No Surprise Act is addressed. Qualifying healthcare entities that the Act affects are reviewed, and in what ways. The author observes and evaluates the insurer, patient, and provider perspectives of the Act, but leans heavily toward agreeing with public health policy. Both providers and insurers, along with other affected entities, are still working towards defining the line of what qualifies as a “reasonable set rate” and which healthcare services should be included and defined further in the No Surprises Act.
Heller, R. et al. [25]	Federal Out-of-Network Balance-Billing Legislation: Context and Implications for Radiology Practices	Radiology	Radiology providers and their professional stakeholder organizations	The authors cite surprise billing legislation as a distributor to the insurer–physician relationship and good-faith negotiations, impacting in-network negotiated contracted rates. Replacing prior good-faith practices, the law is cited as replacing such efforts with arbitration requirements between the parties. Radiology providers are encouraged to participate in stakeholder advocacy to address vague No Surprises Act shortcomings.
Heller, I. et al. [26]	The Surprise Insurance Gap: History, Context, and Proposed Solutions	Journal of the American College of Radiology	Hospital-based radiology providers and patients receiving imaging services	“Surprise billing” is cited as a misnomer because it is properly referred to as a “surprise insurance gap” instead, and such a discrepancy can have serious effects for patients and their families. Hospital-based radiology procedures have been linked to the balance-billing problem for out-of-network imaging providers. The use of benchmarking and alternative dispute resolution are the two most generally proposed ways for determining insurance company reimbursement to providers for out-of-network services. The authors cite any attempts “price set” with a benchmark value as a risk, as setting a predetermined value for services to protect against unexpected costs could have unanticipated and serious implications (such as interrupting good-faith negotiations between insurers and providers and limiting access to treatment). The alternative dispute resolution mechanism can safeguard patients, reduce the number of unexpected out-of-network invoices, and save money.
Hoadley & Lucia [27]	The No Surprises Act: A Bipartisan Achievement to Protect Consumers from Unexpected Medical Bills	Journal of Health Politics, Policy and Law	US healthcare legislators, citizens, and medical providers	Americans are at risk of receiving unexpected medical expenses with out-of-network care. Authors cite various techniques employed in multiple state laws that eventually served as a framework for federal surprise billing legislation. Identifying a method for establishing the amount that an insurer should pay to an out-of-network provider was a challenge, even though there was always a broad consensus among stakeholders for safeguarding consumers during both state and federal deliberations. Financial risks to both the patient and the insurance company are addressed.
Huffman, A. [28]	The Rock, Paper, Scissors Contest of “Surprise” Medical Billing: Nobody Wins, Patients Lose	Annals of Emergency Medicine		A review of social media’s presence over surprise billing is provided. The children’s game of rock, paper, scissors is used as an analogy to describe the continuing problem of surprise billing involving physicians, insurance companies, and patients. Twitter is mentioned as one platform that angry patients used to urge congress to act about the issue. Among the lawmakers working to resolve the issue, US Representative Greg Walden of Oregon openly denounced a series of attack ads funded by private equity firms aimed at undermining his own legislation to prevent surprise billing. Back-to-back advertising by groups representing physicians and insurers stated each side’s displeasure over surprise billing during the Democratic presidential primary debates, even though both helped to establish the dynamic and neither is ready to suffer a financial price to rein it in. Despite states having autonomy, their laws do not apply to approximately 60% of Americans privately insured in “self-insured” health plans. These health plans provided by the employer take on the financial risk to provide benefits.
Kyanko, K. & Busch, S. [29]	Surprise Bills from Outpatient Providers: A National Survey	Journal of General Internal Medicine	55,000 US households with a completion rate of 66%	A survey assessed the proportion of privately insured patients that received outpatient surprise bills, experiences with provider directories, and whether patients obtained reimbursement from their insurer. Overall, 3% (N = 207) received at least one surprise outpatient bill in the last year. Those with fair or poor self-reported health were significantly more likely to experience surprise bills; 12% noted that a provider listed in their insurance directory had either incorrect contact information or did not take their insurance. Of visits to out-of-network providers (N = 654), 39% were associated with surprise bills. Most out-of-network visits (70%) were not reimbursed by the insurer, with no out-of-network coverage and an unmet deductible being the most cited reasons. Surprise bills were no more likely to come from specialists compared with primary care providers. Surprise billings are cited as a system-level failure.
La Forgia et al. [30]	Association of Surprise-Billing Legislation with Prices Paid to In-Network and Out-of-Network Anesthesiologists in California, Florida, and New York: An Eco-nomic Analysis	JAMA Internal Medicine	Florida, California, and New York anesthesiology providers	The authors compared the amounts paid both in-network and out-of-network to anesthesiologist providers before and after the implementation of the No Surprises Act that became effective in January 2022. Data were used from Florida, California, and New York (states that had surprise billing legislation in place prior to the federal legislation); the authors used commercial claims data from both in- and out-of-network settings at the ambulatory and out-patient setting. Overall, the study represented that the billed and paid prices paid to providers dropped significantly after the implementation of the No Surprises Act; this alludes to the transformation of how prices will continue to alter the coming years after this federal legislation has been passed. After legislation was passed in California addressing surprise billing, reductions in price were seen and declines by 13.6%
Long, C. et al. [31]	Understanding Surprise Out-of-Network Billing in Hand and Upper Extremity Care	Journal of Hand Surgery	Hand surgery providers and patients	The field of hand surgery often involves emergency-related surprise billing. Because of the interdisciplinary nature of hand care and the number of ancillary services involved, out-of-network billing can be used at several levels, even if the hand surgeon is in-network for the patient. Surprise billing is assessed in the context of hand surgery, which is poorly understood in hand care—but it is believed that it has a significant impact on the patient population. Essential aspects of surprise billing are addressed and it is critical for the area of hand surgery to better understand the prevalence, operationalization, and policies of surprise billing.
Molyneux, J. [32]	Surprise Medical Bills: Are We Any Nearer to a Federal Fix?	AJN, American Journal of Nursing	Patients and providers undergoing arbitration related to out-of-network healthcare insurance claims	This article described the process of arbitration agreements for reimbursement between insurers and providers to settle balance bills. Arbitration is noted to raise healthcare costs which affects the patients’ bottom line in the dispute of these out-of-network bills, and this article describes the publicity and hyper awareness that these bills began to receive after the pandemic and the associated bills left to be paid.
Pollitz, K. et al. [33]	US Statistics on Surprise Medical Billing	JAMA	Adults across the US who responded to a survey concerning surprise billing and the potential to be balance-billed	US statistics on surprise billing were analyzed and it was found that 41% of insured adults have received a surprise medical bill and 2/3 of adults are worried about being able to afford surprise medical bills. Rates of surprise bills vary by state but, on average, 18% of emergency department visits result in at least one surprise bill in the US.
Reddy, R. & Duffy, E. [34]	Congress Ends Surprise Billing: Implications for Payers, Providers, and Patients	American Journal of Managed Care		Three aspects behind the New bipartisan-supported No Surprises Act are reviewed: the need for federal action, the compromise, and the implications. Authors claim that the implications of the No Surprises Act will negatively affect physician revenues, while increasing pricing transparency, and dropping commercial insurance premiums. The author concludes that this legislation will reduce “competitiveness… reduce consumer cost… [and] add regulatory complexity for some physician practices; for patients, however, this legislation creates a market of fairness and transparency.”
Rha, J. et al. [35]	Markup on Services Provided to Medicare Beneficiaries by Otolaryngologists in 2017: Implications for Surprise Billing	Otolaryngology—Head and Neck Surgery	Otolaryngologists and patients	Authors assessed the potential markup difference between OON commercially insured patients and publicly available Medicare data. A low potential for balance-billing for otolaryngologist providers was established.
Richman, B. et al. [36]	The No Surprises Act and Informed Financial Consent	The New England Journal of Medicine	Healthcare policy/statement analysis	Flaws of the No Surprises Act are addressed. For example, in exchange for bailout funding, the Coronavirus Aid, Relief, and Economic Security (CARES) Act prohibited providers from collecting copayments or pursuing balance bills for coronavirus testing and treatment. Reports indicated that patients still received surprise billings, making it clear that, in the healthcare setting, making a practice illegal will not stop it from happening. The No Surprise Act sets requirements to combat these issues that have outraged many patients. Providers are required to obtain explicit informed consent from each patient regarding the patient’s financial responsibility. It prohibits out-of-network providers from charging patients amounts that exceed the patient’s in-network rates for emergency medical care, air ambulance services, and non-emergency services delivered by out-of-network providers at in-network facilities.
Rosenwald, E. [37]	Rethinking the Emergency-Room Surprise Billing Crisis: Why Are Patients Liable for Emergency Care They Do Not Seek?	Washington University Journal of Law & Policy	Healthcare policy/statement analysis	An imagining of the reality of billing expenses accrued over the stay in the hospital and expenses from surprise billings is reviewed. The purpose of this article is to examine the legislation and policy surrounding emergency patients held liable for the entire cost of their medical bill. This article also gives suggestions about and criticism of policies made for the best interests of the patient.
Scheiman, J. et al. [38]	Surprise billing for colonoscopy: The scope of the problem	Annals of Internal Medicine	Patients undergoing a colonoscopy procedure and receiving surprise medical bills	Out-of-network bills not included in federal mandates incur considerable out-of-pocket costs for patients screening for colorectal cancer. The author estimates the frequency, amount, and source of out-of-network claims for insured patients having a colonoscopy. Findings report that out-of-network claims averaged hundreds of dollars more than the typical insurance bill. One in 12 procedures did not have an association with the screening. Findings suggested that 12.1% of these procedures had out-of-network costs billed to the patient.
White, F. [39]	Surprise Billing in a Hospital Emergency Department—An Ethical, Contractual, and Legislative Conundrum	American Journal of Bioethics	Hospital ethics committee evaluation of physician balance-billing	Ethical responsibilities of a hospital’s ethics committee’s conclusions on whether a physician can set and charge a fee as a third-party reimbursement are examined, yet a method or recommendation is not provided. Legitimate responses for third-party billing under policy and legislation are reviewed. Such responses, while unreasonable for the patient, do not invalidate the ethical principle of transparency or federal provisions.

## Data Availability

Not applicable.

## References

[1] Lieneck C., Darty K., Huddleston C., Kreczmer J., Lambdin S., Young D. (2022). Hospital Price Transparency Perceptions and Observations in the United States: A Rapid Review. Int. J. Acad. Appl. Res..

[2] Hoadley J., O’Brien M., Lucia K. No Surprises Act: A Federal–State Partnership to Protect Consumers from Surprise Medical Bills. https://www.commonwealthfund.org/publications/fund-reports/2022/oct/no-surprises-act-federal-state-partnership-protect-consumers.

[3] Centers for Medicare and Medicaid Services (CMS) Ending Surprise Medical Bills. https://www.cms.gov/nosurprises.

[4] Consumer Financial Protection Bureau What Is a “Surprise Medical Bill” and What Should I Know about the No Surprises Act?. https://www.consumerfinance.gov/ask-cfpb/what-is-a-surprise-medical-bill-and-what-should-i-know-about-the-no-surprises-act-en-2123/.

[5] Hoadley J., Kona M. How States Are Using Independent Dispute Resolution to Resolve Out-of-Network Payments in Surprise Billing. https://www.commonwealthfund.org/blog/2020/how-states-are-using-independent-dispute-resolution-resolve-out-network-payments-surprise.

[6] Keith K., Hoadley J., Lucia K. Federal Officials Revise Approach To Arbitration Under No Surprises Act. https://www.healthaffairs.org/content/forefront/federal-officials-revise-approach-arbitration-under-no-surprises-act.

[7] Berlin J. (2021). Federal Fairness? Congressional Measure Addresses Out-of-Network Payments. Tex. Med..

[8] Berlin J. (2020). Making Billing More Balanced: Congress Considers Surprise Billing Legislation. Tex. Med..

[9] Bernstein J. (2020). Not the Last Word: Surprise Medical Bills are Hardly Charitable. Clin. Orthop. Relat. Res..

[10] Biener A.I., Chartock B.L., Garmon C., Trish E. (2021). Emergency physicians recover a higher share of charges from out-of-network care than from in-network care. Health Aff..

[11] Brown EC F., Trish E., Ly B., Hall M., Adler L. (2020). Out-of-Network Air Ambulance Bills: Prevalence, Magnitude, and Policy Solutions. Milbank Q..

[12] Busch S.H., Kyanko K.A. (2020). Incorrect provider directories associated with out-of-network mental health care and outpatient surprise bills. Health Aff..

[13] Chartock B.L., Adler L., Ly B., Duffy E., Trish E. (2021). Arbitration over out-of-network medical bills: Evidence from New Jersey payment disputes. Health Aff..

[14] Chhabra K.R., Sheetz K.H., McGuire K., Scott J.W., Nuliyalu U., Ryan A.M. (2020). Most patients undergoing ground and air ambulance transportation receive sizable out-of-network bills. Health Aff..

[15] Chhabra K.R., Brown E.F., Ryan A.M. (2021). No More Surprises—New Legislation on Out-of-Network Billing. N. Engl. J. Med..

[16] Chhabra K.R., Sheetz K.H., Nuliyalu U., Dekhne M.S., Ryan A.M., Dimick J.B. (2020). Out-of-Network Bills for Privately Insured Patients Undergoing Elective Surgery with In-Network Primary Surgeons and Facilities. JAMA J. Am. Med. Assoc..

[17] Colla C. (2021). Surprise Billing-A Flashpoint for Major Policy Issues in Health Care. JAMA.

[18] Cooper Z., Scott Morton F., Shekita N. (2020). Surprise! Out-of-Network Billing for Emergency Care in the United States. J. Political Econ..

[19] Duffy E.L., Ly B., Trish E., Adler L. (2021). Commercial and Medicare advantage payment for anesthesiology services. Am. J. Manag. Care.

[20] Duffy E.L., Ly B., Trish E., Adler L. (2020). Policies to address surprise billing can affect health insurance premiums. Am. J. Manag. Care.

[21] Duffy E.L., Adler L., Ginsburg P.B., Trish E. (2020). Prevalence and characteristics of surprise out-of-network bills from professionals in ambulatory surgery centers. Health Aff..

[22] Fuse Brown E.C., McDonald A., Nguyen N.T. (2020). What States Can Do to Address Out-of-Network Air Ambulance Bills. J. Law Med. Ethics.

[23] Fuse Brown E.C. (2020). Stalled Federal Efforts to End Surprise Billing—The Role of Private Equity. N. Engl. J. Med..

[24] Hall M.A. (2021). A “Surprise” Health Policy Legislative Victory. Hastings Cent. Rep..

[25] Heller R.E., Gaines E., Parti N., Duszak R. (2021). Federal Out-of-Network Balance Billing Legislation: Context and Implications for Radiology Practices. Radiology.

[26] Heller IR E., Zaafran S., Gabriel A., Parti N., Richards F. (2020). The Surprise Insurance Gap: History, Context, and Proposed Solutions. J. Am. Coll. Radiol..

[27] Hoadley J., Lucia K. (2022). The No Surprises Act: A Bipartisan Achievement to Protect Consumers from Unexpected Medical Bills. J. Health Politics Policy Law.

[28] Huffman A. (2021). The Rock, Paper, Scissors Contest of “Surprise” Medical Billing: Nobody Wins, Patients Lose. Ann. Emerg. Med..

[29] Kyanko K.A., Busch S.H. (2021). Surprise Bills from Outpatient Providers: A National Survey. J. Gen. Intern. Med..

[30] La Forgia A., Bond A.M., Braun R.T., Kjaer K., Zhang M., Casalino L.P. (2021). Association of Surprise-Billing Legislation with Prices Paid to In-Network and Out-of-Network Anesthesiologists in California, Florida, and New York: An Economic Analysis. JAMA Intern. Med..

[31] Long C., Cho B.H., Giladi A.M. (2021). Understanding Surprise Out-of-Network Billing in Hand and Upper Extremity Care. J. Hand Surg..

[32] Molyneux J. (2020). Surprise Medical Bills: Are We Any Nearer to a Federal Fix?. AJN Am. J. Nurs..

[33] Pollitz K., Lopes L., Kearney A., Rae M., Cox C., Fehr R., Rousseau D. (2020). US Statistics on Surprise Medical Billing. JAMA.

[34] Reddy R., Duffy E.L. (2021). Congress Ends Surprise Billing: Implications for Payers, Providers, and Patients. Am. J. Manag. Care.

[35] Rha J., Rathi V.K., Naunheim M.R., Miller L.E., Gadkaree S.K., Gray S.T. (2021). Markup on Services Provided to Medicare Beneficiaries by Otolaryngologists in 2017: Implications for Surprise Billing. Otolaryngol. Head Neck Surg..

[36] Richman B., Hall M., Schulman K. (2021). The No Surprises Act and Informed Financial Consent. N. Engl. J. Med..

[37] Rosenwald E.S. (2021). Rethinking the Emergency-Room Surprise Billing Crisis: Why Are Patients Liable for Emergency Care They Do Not Seek?. Wash. Univ. J. Law Policy.

[38] Scheiman J.M., Fendrick A.M., Nuliyalu U., Ryan A.M., Chhabra K.R. (2021). Surprise billing for colonoscopy: The scope of the problem. Ann. Intern. Med..

[39] White F.J. (2020). Surprise Billing in a Hospital Emergency Department—An Ethical, Contractual, and Legislative Conundrum. Am. J. Bioeth..

